# COVID-19 and dysnatremia: A comparison between COVID-19 and non-COVID-19 respiratory illness

**DOI:** 10.1177/20503121211027778

**Published:** 2021-06-30

**Authors:** Philip JGM Voets, Sophie C Frölke, Nils PJ Vogtländer, Karin AH Kaasjager

**Affiliations:** 1 Department of Nephrology, Gelre Hospital, Apeldoorn, The Netherlands; 2 Department of Internal Medicine, University Medical Center Utrecht, Utrecht, The Netherlands

**Keywords:** COVID-19, SARS coronavirus, hyponatremia, hypernatremia, dysnatremia

## Abstract

**Objective:**

To investigate the occurrence of disorders of water and sodium balance in COVID-19 in our clinic.

**Methods:**

In this retrospective chart review, patients were included if a polymerase chain test result for SARS-CoV-2 was obtained and if at least one plasma sodium concentration measurement was obtained during the period from March to June 2020. The occurrences of hyponatremia and hypernatremia were compared between 193 SARS-CoV-2-positive and 138 SARS-CoV-2-negative patients. A χ² test was used to determine statistical significance, and the corresponding *p*-values were calculated.

**Results:**

Hypernatremia was significantly more frequently observed in COVID-19 patients, in 38% (74 of 193), versus only 8% in SARS-CoV-2-negative patients (11 of 138) (*p* < 0.01). Hyponatremia was observed in 34% of the included COVID-19 patients (65 of 193) versus 24% of SARS-CoV-2-negative patients (33 of 138). In 12% of all COVID-19 patients (23 of 193), both hyponatremia and hypernatremia were observed at some point during their admission. Among the non-COVID-19 patients, only 4% showed these plasma sodium concentration fluctuations (5 of 138). The mortality rate among the hospitalized COVID-19 patients was 23% (45 of 193). Correcting for double-counting, more than 71% (32 of 45) of the deceased COVID-19 patients developed dysnatremia (hyponatremia, hypernatremia or both) versus 57% (84 out of 148) of the surviving COVID-19 patients.

**Conclusion:**

Disorders of water and sodium balance—and especially hypernatremia—seem to be a common occurrence in COVID-19 patients. This has important implications for the treatment of COVID-19 patients.

## Introduction

The ongoing pandemic known as coronavirus disease 2019 (COVID-19), which is caused by the novel severe acute respiratory syndrome coronavirus 2 (SARS-CoV-2), has swept across the globe in a matter of weeks and has stirred healthcare professionals and governments everywhere to the highest degree.^1,2^ Its impact on everyday life, ranging from social distancing to the widespread ban on social gatherings, is unmistakable and profound. Being a new infectious disease, very little is known about the complications of COVID-19, both short-term and long-term.^1^ Recently, a lot of attention has been garnered by the remarkably high incidence of pulmonary edema and pulmonary embolisms in COVID-19 patients as opposed to similar (viral) respiratory tract infections.^3,4^ A recent meta-analysis in 24,410 COVID-19 patients showed that their predominant symptoms were fever (78%), cough (56%), and fatigue (31%), with 19% of all hospitalized patients requiring non-invasive ventilation and 9% requiring invasive ventilation.^2^ A growing body of evidence shows that COVID-19 is also accompanied by several extra-pulmonary phenomena, such as disorders of the water and sodium balance.^5–7^

In this report, we have investigated the occurrence of dysnatremia in COVID-19 patients compared to non-COVID-19 respiratory illness, rather than healthy controls, based on data from the patient database of the University Medical Center Utrecht (UMCU). By comparing the results of COVID-19 patients with the results of other respiratory illnesses, we were better able to determine whether plasma sodium outcomes should be considered “COVID-19-specific” or are rather the result of respiratory illness in a broad sense. We believe that the results of our retrospective chart review will help raise clinical awareness in every physician treating COVID-19 patients, especially now that the world is coping with this ongoing pandemic.

Below, we discuss our findings.

## Methods

For this retrospective chart review, we have used patient data collected for the COvid-19 PAtients CHaracteristics (COVPACH) study, which has registered the laboratory test results of all hospitalized COVID-19 patients in UMCU, including patients admitted to the intensive care unit (ICU), during the period March to June 2020. Ethical review was waived by the Medical Ethical Committee (MEC) of UMCU (MEC reference number: 20-284). Consent was obtained using an opt-out procedure, in accordance with the hospital guidelines and with approval of the institutional research board. The medical records of these patients were accessed anonymously and none of the authors were their treating physicians. Patients were included in our study if the following two conditions were met:^1^

1. A polymerase chain reaction (PCR) test result for SARS-CoV-2 was obtained.
2. At least one—but preferably more than one—plasma sodium concentration measurement was obtained (at any time during admission). For every included patient, his or her medical records were checked for additional biochemical parameters, such as plasma osmolality, urine osmolality, and urine sodium concentration. Hypernatremia was defined as a plasma sodium concentration of 146 mmol/L or above, and hyponatremia was defined as a plasma sodium concentration of 134 mmol/L or below. In total, 331 hospitalized patients with clinical suspicion of COVID-19 were screened via a SARS-CoV-2 PCR test. A total of 193 patients tested positive for SARS-CoV-2. The 138 patients who tested negative but were exhibiting the symptoms of a non-COVID-19 respiratory tract disease (such as a bacterial pneumonia or a non-COVID-19 viral respiratory tract infection) were used as a control group to compare the occurrence of dysnatremia in patients with COVID-19 and those with another respiratory illness. Finally, in order to analyze the association between dysnatremia and mortality in COVID-19, the occurrences of dysnatremia (corrected for double-counting) in deceased patients and patients who stayed alive were compared. Both patient groups were compared in terms of mean age, gender, body mass index (BMI), blood pressure, plasma creatinine concentration, inflammatory biomarkers, relevant comorbidity, and immunosuppressant medication use. These results are summarized in Table 1.

**Table 1.** Characteristics of patients in COVID-19 group and control group.

Mean baseline characteristics	COVID-19 group (*n* = 193)	Control group (*n* = 138)
Gender: M/F (%)	60.2/39.8	55.3/44.7
Age (years)	64.3	61.7
Body mass index (kg/m^2^)	28.5	26.2
Systolic blood pressure (mmHg)	138	137
Diastolic blood pressure (mmHg)	73	79
Plasma creatinine level (μmol/L)	120.8	98.3
Plasma C-reactive protein level (mg/L)	147	87
Hypertension (%)	37	14
Diabetes mellitus (%)	21	4
Immunosuppressant medication use (%)	18	9

### Statistical analysis

A χ² test was used to determine statistical significance, and the corresponding *p*-values were calculated. A statistical power analysis was performed in order to evaluate the required patient group sizes.

### Ethical conflict

The authors declare to have no ethical conflict.

## Results

Hypernatremia was observed in 38% of the included COVID-19 patients (74 of 193) versus in only 8% of the SARS-CoV-2-negative patients (11 of 138), which is a strong significant difference (*p* < 0.01). On several occasions, the plasma sodium concentration in these patients reached critical values as high as 174 mmol/L. The obtained spot urine samples of these hypernatremic patients showed urine osmolalities ranging from 509 to 819 mOsmol/L, with an average urine osmolality of 604 mOsmol/L. Additional analysis showed that hypernatremia in COVID-19 patients occurred significantly more frequently during ICU admission than outside the ICU (*p* < 0.01). Our calculations showed that using a two-sided test, a 5% significance level (α = 0.05), and a statistical power of 80% (β = 0.20), a minimum sample size of 27 per group (*n* = 54) was required to detect this difference. Therefore, our group size was sufficiently large.

Hyponatremia was observed in 34% of the included COVID-19 patients (65 of 193) versus in 24% of the SARS-CoV-2-negative patients (33 of 138). This difference, while indicative of a COVID-19-specific effect, did not reach statistical significance at a cut-off *p*-value of <0.01 (*p* = 0.06). Although hyponatremia turned out to be a common phenomenon among COVID-19 patients, it also turned out to be relatively mild. In none of these patients did the plasma sodium concentration drop below 127 mmol/L. The obtained spot urine samples of these hyponatremic patients showed urine osmolalities ranging from 274 to 598 mOsmol/L and urine sodium concentrations ranging from 11 to 207 mmol/L. Their average urine osmolality was 432 mOsmol/L and their average urine sodium concentration was 50 mmol/L.

Interestingly, in 12% of all COVID-19 patients (23 of 193), both hyponatremia and hypernatremia were observed at some point during their admission, with some patients even displaying frequent fluctuations of their plasma sodium concentration. Among the non-COVID-19 patients with a respiratory illness, only 4% showed these plasma sodium concentration fluctuations (5 of 138). Taking the aforementioned results together—and counting the 23 COVID-19 patients who developed both hyponatremia and hypernatremia during their admission as one case of dysnatremia in order to prevent double-counting—60% of all COVID-19 patients (65 plus 74 minus 23 = 116 of 193) developed dysnatremia, defined as hyponatremia, hypernatremia, or both, versus 28% of the patients in the control group (33 plus 11 minus 5 = 39 of 138). Dysnatremia occurred significantly more in COVID-19 patients than in patients who were tested negative (*p* < 0.01).

The mortality rate among the hospitalized COVID-19 patients from the COVPACH study turned out to be 23% (45 of 193). Dysnatremia was observed in little more than 71% of the deceased COVID-19 patients (32 of 45) versus almost 57% (84 of 148) of the COVID-19 patients who lived, once again correcting for double-counting, but this difference did not reach statistical significance at a cut-off *p*-value of <0.01.

The main results of this study are summarized in Table 2.

**Table 2.** Summary of results.

Outcome	COVID-19 group (*n* = 193) (%)	Control group (*n* = 138) (%)	*p*-value^a^
Hypernatremia	74/193 (38)^b^	11/138 (8)	<0.01
Hyponatremia	65/193 (34)	33/138 (24)	0.06
Dysnatremia	116/193 (60)	39/138 (28)	<0.01
In deceased patients^c^	32/45 (71)	4/6 (67)	
In surviving patients^c^	84/148 (57)	35/132 (27)	

ICU: intensive care unit.

a A χ² test was used to determine statistical significance.

b Hypernatremia in COVID-19 patients occurred significantly more frequently during ICU admission than outside the ICU (*p* < 0.01).

c The difference in the occurrence of dysnatremia between deceased and surviving COVID-19 and non-COVID-19 patients did not reach statistical significance.

## Discussion

In this study, we have presented the results of the retrospective chart review in which we have investigated the occurrence of hypernatremia among 193 SARS-CoV-2-positive compared to 138 clinically suspected but SARS-CoV-2-negative patients from our clinic. In our opinion, comparing the COVID-19 patients to patients with a non-COVID-19 respiratory illness, rather than comparing them to healthy controls (i.e. those without afflicted respiratory systems), was indicative of the COVID-19-specific biochemical characteristics and not simply of the characteristics of respiratory illness in a broader sense. Our findings clearly demonstrate that disorders of water and sodium balance are a common—but probably underreported—phenomenon in COVID-19, with 38% of the aforementioned patients developing hypernatremia during their admission, 34% developing hyponatremia, and a total of 60% developing some form of dysnatremia (hyponatremia, hypernatremia, or both, correcting for double-counting). Among the 138 patients who showed signs of COVID-19, but were found to be SARS-CoV-2-negative, hypernatremia, hyponatremia, and dysnatremia occurred in 8%, 24%, and 28%, respectively. The fact that 12% of all analyzed COVID-19 patients developed both hyponatremia and hypernatremia during their hospital admission—without a noted temporal relationship with any administered intravenous fluids—might reflect the severity of COVID-19 on a cellular level. In many ways, this loss of the human body’s ability to maintain electrolyte homeostasis is reminiscent of the putative ‘sick cell syndrome’ that can be observed in critically ill or terminal patients in the intensive care wards (supposedly as a result of sodium-potassium antiporter dysfunction due to intracellular ATP depletion). One could even hypothesize that dysnatremia could be considered an (early) indicator of impending bodily imbalance and exhaustion in a broad sense.

Interestingly, whereas almost two of every five COVID-19 patients developed hypernatremia, this occurred in only 8% of the non-COVID-19 respiratory illness patients. This finding contradicts earlier work by—among others—Atila et al.,^5^ who found that the occurrence of hypernatremia was comparable among their COVID-19 group and control group, and Hirsch et al.,^6^ who found a compound hypernatremia prevalence of only 7% (3.2% for mild hypernatremia, defined as a plasma sodium concentration between 145 and 149 mmol/L, and 3.8% for severe hypernatremia, defined as a plasma sodium concentration of 150 mmol/L or above) in a large cohort of 9946 COVID-19 patients. Similar hypernatremia prevalence numbers have been reported by other authors.^7,8^ However, we have not found any relevant difference between the Dutch approach to COVID-19 and COVID-19 treatment in other countries. Based on the appropriately concentrated urine in our group of hypernatremic COVID-19 patients (with an average urine osmolality of 604 mOsmol/L), dehydration seems to be a very plausible explanation for their hypernatremia.^9^ It seems likely that the reluctance of many physicians to initiate intravenous fluid therapy due to the risk of (an exacerbation of) bradykinin-mediated pulmonary edema—which has generally been considered a hallmark of COVID-19—in these bedridden patients with a limited access to drinking water might be an important contributory factor in the development of the observed (and probably iatrogenic) hypernatremia.^4^ The direct manipulation of the renin–angiotensin–aldosterone system (RAAS) by binding of SARS-CoV-2 to angiotensin-converting enzyme 2 (ACE2) has also been implicated in the pathogenesis of COVID-19-related electrolyte disturbances.^8^ In some cases, the relative disinclination of nurses, nutrition assistants, and other healthcare workers to enter the isolation rooms of COVID-19 patients due to the stringent and time-consuming personal protective measures might have added to this frequent occurrence of hypernatremia. Our finding that the development of hypernatremia in COVID-19 patients during ICU admission is higher than outside the ICU supports the hypothesis of hypernatremia due to iatrogenic dehydration.^8^ The use of dexamethasone to ameliorate the hyperinflammation that can accompany COVID-19 seems to be another contributing factor.^1^ Another plausible explanation for the frequent occurrence of hypernatremia in COVID-19 patients in the ICU could be osmotic urea diuresis, which is a prime example of why the solute-free water clearance (which is negative in these patients due to the massive urinary urea excretion) should be considered misleading with regard to the analysis of dysnatremia.^10^ The much more accurate electrolyte-free water clearance (which is positive in these patients due to the relatively low concentration of electrolytes in their urine) easily explains the development of hypernatremia.^10,11^ As presented in the results, the plasma sodium concentration can reach alarming heights. Future studies should focus on the balance between dehydration prevention during ICU admission and avoiding pulmonary edema in COVID-19.

Hyponatremia was frequently observed in both COVID-19 patients and patients with a non-COVID-19 respiratory illness, 34% and 24%, respectively. This hyponatremia prevalence in COVID-19 patients is comparable to previously published results.^5,6^ In comparison, a large population-based cross-sectional National Health and Nutrition Examination Survey (NHANES) study from 2013 showed that the prevalence of hyponatremia among 14,697 healthy US adults was approximately 1.7%.^12^ In line with a small number of previously published reports, the syndrome of inappropriate antidiuretic hormone release (SIADH) seems to be the primary cause of COVID-19-associated hyponatremia, based largely on the original diagnostic criteria as proposed by Bartter and Schwartz, namely, hypotonic hyponatremia with inappropriately concentrated urine (the average urine osmolality of 432 mOsmol/L suggests inappropriate release of antidiuretic hormone (ADH) in the context of hyponatremia) and euvolemia (in the absence of diuretic use, the average urine sodium concentration of 50 mmol/L suggests a lack of RAAS activation).^13,14^ As pulmonary disease is a fairly common cause of SIADH, this seems pathophysiologically plausible.^11^ This being said, hypovolemia-mediated ADH release could also have played an etiological role in a minority of cases in which the urine sodium excretion was strongly reduced (<15 mmol/L), especially because many physicians have been reluctant to initiate intravenous fluid therapy in these patients due to the fear of pulmonary edema. As the urine osmolality in none of the hyponatremic COVID-19 patients dropped below 274 mOsmol/L, other factors, such as ‘tea and toast syndrome’, seem less relevant in COVID-19-associated hyponatremia, even though the nutritional status of these patients is often questionable at best.^11^

A limitation of our study is that—while the occurrence of dysnatremia during admission could be reliably established in our patient groups—our data often did not allow the analysis of a temporal relationship between the onset of dysnatremia and the clinical course of COVID-19, which would have been desirable for proving causality. Furthermore, due to a large number of confounders, such as—but certainly not limited to—patient age, smoking status, and co-morbidity, the possible causality of dysnatremia in COVID-19-related deaths could not be reliably established in our retrospective cohort of COVID-19 patients. Although a more rigorous and definitive analysis remains desirable on this issue, our data do suggest that dysnatremia occurs more frequently in patients succumbing to COVID-19 (71%) than in those surviving COVID-19 (57%). This is in line with previously published analyses, in which dysnatremia has consistently been found to be an independent risk factor for mortality in COVID-19 patients.^6,7,15,16^ It seems reasonable to assume that this observation can be reliably extrapolated to COVID-19 patients.^6,7,15,16^ As discussed above, failure to maintain water and sodium homeostasis might be indicative of impending death in COVID-19 patients, regardless of the causality. We also would like to stress that all the analyzed COVID-19 patients in our study were hospitalized; our results should not be blindly extrapolated to milder cases of COVID-19 which do not require hospitalization. Finally, we have included patients in either the COVID-19 group or the non-COVID-19 (control) group based on the results of their SARS-CoV-2 PCR tests. We realize that perhaps some PCR-negative COVID-19 patients have inadvertently been included in the control group. However, since the diagnosis of PCR-negative COVID-19 is notoriously difficult and its definition relatively vague, we feel justified in this approach although it could lead to an underestimation of our results.

In conclusion, disorders of water and sodium balance—and especially hypernatremia, contrary to the findings of several previously published studies—seem to be a very common extrapulmonary occurrence in COVID-19 patients and are associated with an increase in morbidity and probably even mortality. In this retrospective chart review, we have attempted to raise awareness of this potentially dangerous complication of COVID-19, which might even reflect its severity. Hopefully, this will have appropriate implications for the treatment of and care for COVID-19 patients now that the world is coping with this ongoing pandemic.
